# Establishing initial auditory‐visual conditional discriminations and emergence of initial tacts in young children with autism spectrum disorder

**DOI:** 10.1002/jaba.586

**Published:** 2019-06-06

**Authors:** Wayne W. Fisher, Billie J. Retzlaff, Jessica S. Akers, Andresa A. DeSouza, Ami J. Kaminski, Mychal A. Machado

**Affiliations:** ^1^ University of Nebraska Medical Center's Munroe‐Meyer Institute; ^2^ University of Alaska Anchorage

**Keywords:** auditory‐visual conditional discrimination, autism spectrum disorder, differential observing response, emergent responding, identity‐match prompt, receptive‐expressive transfer

## Abstract

Children with autism spectrum disorder (ASD) often display impaired listener skills, and few studies have evaluated procedures for establishing initial auditory‐visual conditional discrimination skills. We developed and evaluated a treatment package for training initial auditory‐visual conditional discriminations based on the extant research on training such discriminations in children with ASD with at least some preexisting skills in this area. The treatment package included (a) conditional‐only training, (b) prompting the participant to echo the sample stimulus as a differential observing response, (c) prompting correct selection responses using an identity‐match prompt, (d) using progressively delayed prompts, and (e) repeating trials until the participant emitted an independent correct response. Results indicated all participants mastered all listener targets, and the two participants for whom we tested the emergence of corresponding tacts showed mastery of most tacts without direct training. We discuss these results relative to prior research on listener skills and tacts.

Responding effectively to the words and sentences spoken by others (called listener or receptive‐language skills or auditory comprehension) is critical to early child development, literacy, and day‐to‐day functioning (Biemiller & Slonim, 2001; Hart & Risley, 1995; McCarthy, 1954; Nelson, 1973). Children with autism spectrum disorder (ASD) display marked deficits in this area of development (Rapin & Dunn, 1997). For some children with ASD, listener skills are so impaired that the parents question whether their child is deaf, and diagnosticians categorize the deficit as verbal auditory agnosia (or word deafness; Tuchman, Rapin, & Shinnar, 1991). This pattern suggests that many children with ASD do not acquire important listener skills through typical social contingencies in the natural environment (e.g., a parent labeling and pointing to body parts and praising the child for imitating their sounds or actions). As such, it is not surprising that almost all early intensive behavioral intervention curricula emphasize training listener skills (e.g., Leaf & McEachin, 1999; Lovaas, 1981, 2003; Maurice, Green, & Luce, 1996; Sundberg, 2008; Sundberg & Partington, 1998).

An early and important listener skill taught to children with ASD is auditory‐visual conditional discrimination, which typically involves the child learning to select the correct comparison picture following a vocal discriminative stimulus (S^D^). For example, when presented with an array of pictures (e.g., apple, banana, kiwi) and a spoken word corresponding to one of the pictures (e.g., “apple”), the correct response would be to touch or point to the picture corresponding to the spoken sample stimulus (i.e., the picture of the apple). These listener skills are important for navigating the natural environment. For example, a caregiver may say, “Bring me the apple” to a child while packing lunch for the day, and the child must respond in accordance with the spoken instruction. Young children with ASD show impaired acquisition of this type of listener behavior, and a substantial amount of recent research has focused on how to train auditory‐visual conditional discriminations in this population (see Grow & LeBlanc, 2013, and LaMarca & LaMarca, 2018, for focused reviews).

Given the importance of training auditory‐visual conditional discriminations, a critical question for the field is, “What procedure or combination of procedures should be used to train initial auditory‐visual conditional discriminations in children with ASD?” For example, MacDonald and Langer (2018) published a comprehensive guide for teaching discrimination skills that recommends progressing from teaching simple discriminations to more complex discriminations such as auditory‐visual conditional discriminations. Unfortunately, the question of how to teach initial auditory‐visual conditional discriminations is difficult to answer with confidence from the current literature on training such discriminations because almost all of the participants in the extant literature have been individuals with at least some preexisting auditory‐visual conditional discrimination repertoire (Carp, Peterson, Arkel, Petursdottir, & Ingvarsson, 2012; Clark & Green, 2004; Delfs, Conine, Frampton, Shillingsburg, & Robinson, 2014; Fisher, Kodak, & Moore, 2007; Grow, Carr, Kodak, Jostad, & Kisamore, 2011; Grow, Kodak, & Carr, 2014; Gutierrez et al., 2009; Holmes, Eikeseth, & Schulze, 2015; Isenhower, Delmolino, Fiske, Bamond, & Leaf, 2018; Kodak et al., 2015; Kodak, Fisher, Clements, Paden, & Dickes, 2011; Leaf, Alcalay, et al., 2016; Leaf, Cihon, et al., 2016; Leaf, Sheldon, & Sherman, 2010; McGee, Krantz, Mason, & McClannahan, 1983; Vedora, & Grandelski, 2015; Williams, Perez‐Gonzalez, & Queiroz, 2005). For example, all of the participants in the Grow et al. (2011, 2014) studies had prior exposure to auditory‐visual conditional discrimination training, and the participants in Holmes et al. (2015) had acquired 100 to 200 such conditional discriminations prior to participating in the investigation.

A careful examination of the participant descriptions in the above‐referenced 17 studies on training auditory‐visual conditional discriminations in children with ASD revealed that just 3 of 57 participants with ASD (5.3%) would be classified as novice learners in this area of development. We defined novice learners as those (a) not mastering any prior auditory‐visual conditional discriminations through training or (b) obtaining a standard score of 20 (i.e., the lowest possible score) on the Peabody Picture Vocabulary Test (PPVT; Dunn & Dunn, 2007), a standardized measure of receptive vocabulary skills. Of the three children in the literature meeting at least one of these criteria: (a) none acquired auditory‐visual conditional discriminations with a point prompt and reinforcement (Victor and Andrew in Kodak et al., 2011; Chris in Carp et al., 2012); and (b) one of them (Chris in Carp et al., 2012) acquired auditory‐visual conditional discriminations with a picture prompt. Given the few novice learners in the extant literature, and their inconsistent responses to intervention, additional research should identify treatment procedures that consistently facilitate initial acquisition of such discriminations.

Our main purpose for the current investigation was to identify and evaluate a set of procedures that, based on the extant literature, could potentially result in consistent acquisition of auditory‐visual conditional discriminations in novice learners with ASD (Experiment 1). We reasoned that combining individual procedures reported in the literature to facilitate acquisition in a broader group of individuals with ASD, into a treatment package, might be more successful at establishing auditory‐visual conditional discrimination in novice learners with ASD than any single procedure used in isolation. Therefore, we used the extant literature to select the following components for our treatment package.

First, we presented learning trials in the conditional‐only trial format described by Grow et al. (2011, 2014), because they showed that this format produced acquisition of auditory‐visual conditional discrimination to mastery levels more consistently than the simple‐conditional format. Second, we included a differential observing response (DOR) in which the participant echoed the spoken sample stimulus (e.g., therapist said, “banana,” and prompted the participant to echo, “banana”) in order to ensure that the participant heard each sample stimulus and discriminated it from the other sample stimuli. Prior research has shown that including a DOR for the sample stimulus can facilitate acquisition of auditory‐visual conditional discriminations (Constantine & Sidman, 1975; Dube & McIlvane, 1999; Geren, Stromer, & Mackay, 1997). Third, we used a progressively delayed prompting procedure to transfer stimulus control from the controlling prompt to each sample stimulus because it often produces relatively rapid acquisition with few errors and relatively low levels of problem behavior (Green, 2001; Grow & LeBlanc, 2013; Sidman & Stoddard, 1967; Touchette, 1971; Walker, 2008; Weeks & Gaylord‐Ross, 1981). Fourth, we selected an identity‐match prompt (i.e., a picture prompt that is identical to the correct comparison stimulus) as the controlling prompt, because it ensures that the individual discriminates the correct comparison stimulus from the other comparison stimuli, and research has shown it to be more effective than a point prompt (Carp et al., 2012; Fisher et al., 2007; Jones & Zarcone, 2014; Vedora & Barry, 2016). Fifth, we included an error‐correction procedure that involved repeating trials on which the participant emitted an error until the participant emitted an independent correct response for that trial (e.g., Schumaker & Sherman, 1970).

This error‐correction procedure ensured that the participant contacted reinforcement for independent correct responses, which may help to prevent or mitigate prompt dependence. Prior research has shown that this error‐correction procedure, sometimes called a *remedial trial* or *repeat until independent*, can have two functions: (a) a stimulus‐control function in the form of increased exposure to sample‐comparison pairings and (b) a negative‐reinforcement function in the form of avoidance of remedial trials contingent on increased independent correct responses (Rodgers & Iwata, 1991; Worsdell et al., 2005). Moreover, Carroll, Joachim, St. Peter, and Robinson (2015) found repeating trials until the participant emitted an independent correct response to be about equally effective as three other commonly used error‐correction procedures, and it proved to be the most efficient for three of five participants and the second most efficient for the remaining two participants. Finally, our procedure differed somewhat from these prior trial‐repetition procedures in that we delivered reinforcement for both prompted and independent correct responses until the participant emitted independent correct responses on 44% of trials, and then we discontinued reinforcement for prompted correct responses. We did this to ensure that participants did not experience lengthy periods without reinforcement during the initial portion of training.

Our second purpose for the current study was to provide a preliminary test as to whether our treatment package for establishing initial auditory‐visual conditional discriminations in novice learners with ASD might also facilitate the emergence of a corresponding tact repertoire in individuals without any preexisting tacts (Experiment 2). That is, we aimed to determine, for example, whether a participant would learn to tact “apple,” “kiwi,” and “banana” as the child's first tacts after learning to respond conditionally as a listener to those same three verbal stimuli spoken by another person. Consistent with the naming hypothesis (Horne & Lowe, 1996), we hypothesized that prompting and requiring participants to echo the sample stimulus (e.g., saying, “apple” after the therapist said, “apple”) and then providing reinforcement for selecting the correct comparison stimulus might also reinforce echoic‐listener relations and thereby facilitate the emergence of tacts (Miguel & Petursdottir, 2009; Petursdottir & Carr, 2011). Therefore, in Experiment 2, we conducted tact probes during baseline and during the maintenance phase to assess whether training audio‐visual conditional discriminations in this manner resulted in the emergence of corresponding tacts in two participants with no prior tact repertoires.

## GENERAL METHOD

### *Participants*

We recruited four children diagnosed with ASD to participate in this evaluation. All four children received services at a university‐based early intervention program. Each had an age equivalent of less than 2 years and a standard score of 20 on the PPVT‐4, and none of them scored higher than chance levels on Level 1 of the PPVT‐4 (all *p*‐values > .25). A trained technician, under the supervision of a licensed psychologist, administered the PPVT‐4 using the procedures detailed in the administration manual. In an attempt to maintain the participant's motivation, we provided reinforcement on a variable ratio (VR) 2 schedule for appropriate behaviors that were not related to the assessment questions (e.g., sitting correctly, attending to therapist) during the administration. Maria and Marco made selections during all trials of the PPVT‐4; however, Pierre only responded on 2 of the 12 trials, and Colton never made a selection during the PPVT‐4.

Prior to beginning the study, all four participants could engage in (a) echoic responses, (b) identity matching with pictures, and (c) emit a small number of single‐word mands. According to caregiver and therapist report, as well as participants' early‐intervention records, none of the participants had a tact repertoire prior to the study. Maria, a 4‐year‐old female who received 3 hr of services per week, and Pierre, a 3‐year‐old male who received 20 hr of service per week participated in Experiment 1. Marco, a 5‐year‐old male who received 20 hr of services per week, and Colton, a 4‐year‐old male who received 9 hr of services per week, participated in Experiments 1 and 2.

### *Setting and Materials*

Sessions occurred in a small cubicle (2.5 m x 2.5 m) in an early‐intervention clinic. Each cubicle contained a table, chairs, program stimuli, data sheets, pen, a timer, and reinforcers (edible items for Maria, iPad® for Pierre, and both edible items and an iPad® for Marco and Colton). Therapists from the early intervention program nominated items which generally appeared to motivate the participant or reported items identified in prior systematic preference assessments conducted in the clinic. We conducted a brief preference assessment (DeLeon et al., 2001) prior to each session for Marco and Colton. We used the same reinforcers across phases (i.e., baseline, treatment, and maintenance) for each participant. Program stimuli consisted of 12.7 cm x 15.2 cm colored pictures of each target.

### *Response Measurement for Experiment 1*

Observers scored correct responses during auditory‐visual discrimination tasks as independent or prompted for each stimulus set. Observers scored a correct *independent* response if the participant touched the target picture following the presentation of the vocal S^D^ prior to the introduction of the identity‐match prompt. Observers scored a *prompted* response if the participant touched the target picture within 5 s of the presentation of the identity‐match prompt. Observers scored incorrect responses if the participant either (a) touched one of the nontarget pictures or (b) did not touch any of the pictures within the allotted time (i.e., time before the trial ended during baseline or time before the next prompt in the prompting sequence during treatment). For Pierre, observers scored independent scanning if he oriented his eyes to each of the comparison stimuli for a least 1 s (Kodak et al., 2011).

Observers also scored whether the participant engaged in the DOR of echoing the S^D^. We defined this response as the participant repeating the vocal S^D^, or engaging in a recognizable approximation of the S^D^ (e.g., saying “wag” for wagon), within 5 s of the first or second presentation of the vocal S^D^. For each session and dependent variable, we calculated the percentage of trials in which the target response occurred by dividing the total number of target responses in a session by the total number of trials (i.e., nine) and then converting the resulting quotient to a percentage.

### *Response Measurement Specific to Experiment 2*

In Experiment 2, observers also recorded whether the participant emitted a correct or incorrect tact during the tact probes. Observers scored a correct tact if the participant emitted the corresponding vocal response within 5 s of the therapist presenting the picture and saying, “What's this?”. Observers scored an incorrect tact if the participant emitted a vocalization that did not correspond to the picture presented by the therapist. Finally, observers scored no response if the participant did not emit a vocal response within 5 s of the S^D^. Similar to Experiment 1, observers counted both incorrect tacts and no responses as errors.

### *Interobserver Agreement (IOA) and Procedural Integrity*

A second, independent observer collected data for at least 30% of sessions in baseline and treatment. We scored an exact agreement for a given response in a given trial if each of the data collectors agreed on whether (a) the response was independent, prompted, incorrect, or no response; (b) the participant engaged in the DOR; and (c) the participant emitted a correct or incorrect tact or no response during the tact probe (Marco and Colton only). For each session and target response, we summed the number of exact agreements, divided that sum by the number of trials (i.e., nine), and converted the quotient to a percentage. For each participant and dependent variable, mean agreement coefficients equaled or exceeded 97.5% (range for individual sessions, 75% ‐ 100%). For Pierre, we also calculated IOA on scanning for 36% of sessions, and the agreement coefficient equaled 100%.

The second observer also collected data on procedural integrity for 13% of Maria's sessions, 5% of Pierre's sessions, 33% of Marco's sessions, and 28% of Colton's sessions. For each session, the observer scored whether the therapist correctly delivered all prompts and consequences for each of the nine trials. That is, for each trial, the observer scored a correct prompt only if the therapist correctly delivered all prompts for that trial (including prompts during error‐correction trials) and a correct consequence only if the therapist delivered all consequences correctly (including consequences during error‐correction trials). We then summed the total correct prompts and consequences for a session, divided that sum by 18, and converted the quotient to a percentage. Across participants, average procedural integrity coefficients equaled or exceeded 99.8% (range for individual sessions, 88.9% ‐ 100%).

### *Experimental Design*

Three of the four participants learned nine receptive‐identification targets (i.e., three sets of three targets, see Table 1). Colton, the fourth participant, learned six targets (i.e., two sets of three targets). We implemented a concurrent multiple‐baseline design across target sets to evaluate the effectiveness of the training procedure for each participant.

**Table 1:** Target Stimuli

Participant	Set	Targets
Maria	1	Whistle, plant, boot
	2	Desk, neck, gate
	3	Goat, plate, candle
Pierre	1	Dog, hat, red
	2	Wagon, eggs, puzzle
	3	Money, bell, clock
Marco	1	Doll, drinking, mittens
	2	Key, lion, sleeping
	3	Door, money, pants
Colton	1	Boat, door, juice
	2	Bee, coat, train

### *Procedure*

#### *Stimulus identification*

Prior to assigning stimuli to sets, we selected and pretested 15 potential target stimuli that were selected from a list of 100 potential targets in a common first‐words list based on the participant being able to echo or emit an approximation of the target. During each pretest session, the therapist presented one trial of each of the 15 stimuli. The therapist placed an array of three stimuli in front of the participant and presented the vocal S^D^ (e.g., “ball”). After each response or 5‐s period with no response, the therapist removed the stimuli and presented the next trial. The therapist did not deliver any programmed consequences following correct or incorrect responses, but did provide reinforcement on a VR 2 schedule for other appropriate behaviors (e.g., sitting correctly, attending to therapist) to maintain participant motivation.

After five pretest sessions, nine targets were selected for the treatment evaluation for each participant except for Colton as indicated above. We excluded stimuli the participant correctly identified on more than 40% of total presentations during the pretest (which rarely occurred). We then ranked the remaining stimuli according to the percentage correct during the pretest (i.e., those correct on 40% of trials, followed by those correct on 20% of trials, followed by those correct on 0% of trials). We matched the assignment of stimuli to each set such that each set had approximately an equivalent number of stimuli the participant correctly selected on 40% of trials, 20% of trials, and 0% of trials. Table 1 displays the stimulus sets for each participant.

### *General Procedures for Experiment 1*

All sessions consisted of nine trials. The therapist placed the three stimuli on the table in front of the participant (on the right, middle, and left) and provided the vocal S^D^ (e.g., “whistle”). The therapist counterbalanced the positioning of the comparison stimuli so that each stimulus appeared in the right, the left, and the middle position three times in each nine‐trial session.

#### *Baseline*

The therapist provided no programmed consequences for correct or incorrect responses and removed the comparison stimuli after the participant selected a picture or 5 s elapsed without a response, whichever came first. The therapist provided reinforcement (e.g., praise and a preferred item) on a VR 2 schedule for appropriate behaviors other than the target response (e.g., sitting correctly, attending to therapist).

#### *Treatment*

The therapist conducted the treatment sessions in a manner identical to baseline sessions except for the following changes. First, to add the echoic DOR on each trial, the therapist placed the comparison stimuli on the table in front of the participant, gently blocked the participant's hands so he or she would not touch the cards, and presented the vocal S^D^ (e.g., “whistle”). If the participant independently echoed the vocal S^D^ within 5 s of the presentation, the therapist removed their hand and allowed the participant to make a selection. If the participant did not echo the target within 5 s, the therapist provided an additional vocal prompt (e.g., “say, whistle”). Following the second vocal prompt, the therapist allowed a selection response, regardless of the participant's response to the vocal prompt. If the participant engaged in an independent correct selection response, the therapist provided descriptive praise (e.g., “Good job touching the apple”) and access to an edible item (Maria, Marco, and Colton) or iPad® (Pierre, Marco, and Colton) for 20 s. If the participant selected the incorrect comparison stimulus or the prompt‐delay interval elapsed, the therapist held up a picture identical to the correct comparison stimulus (i.e., identity‐match prompt). The therapist held up the identity‐match prompt in a neutral location (i.e., not aligned with any of the comparison stimuli).

**Figure 1:**
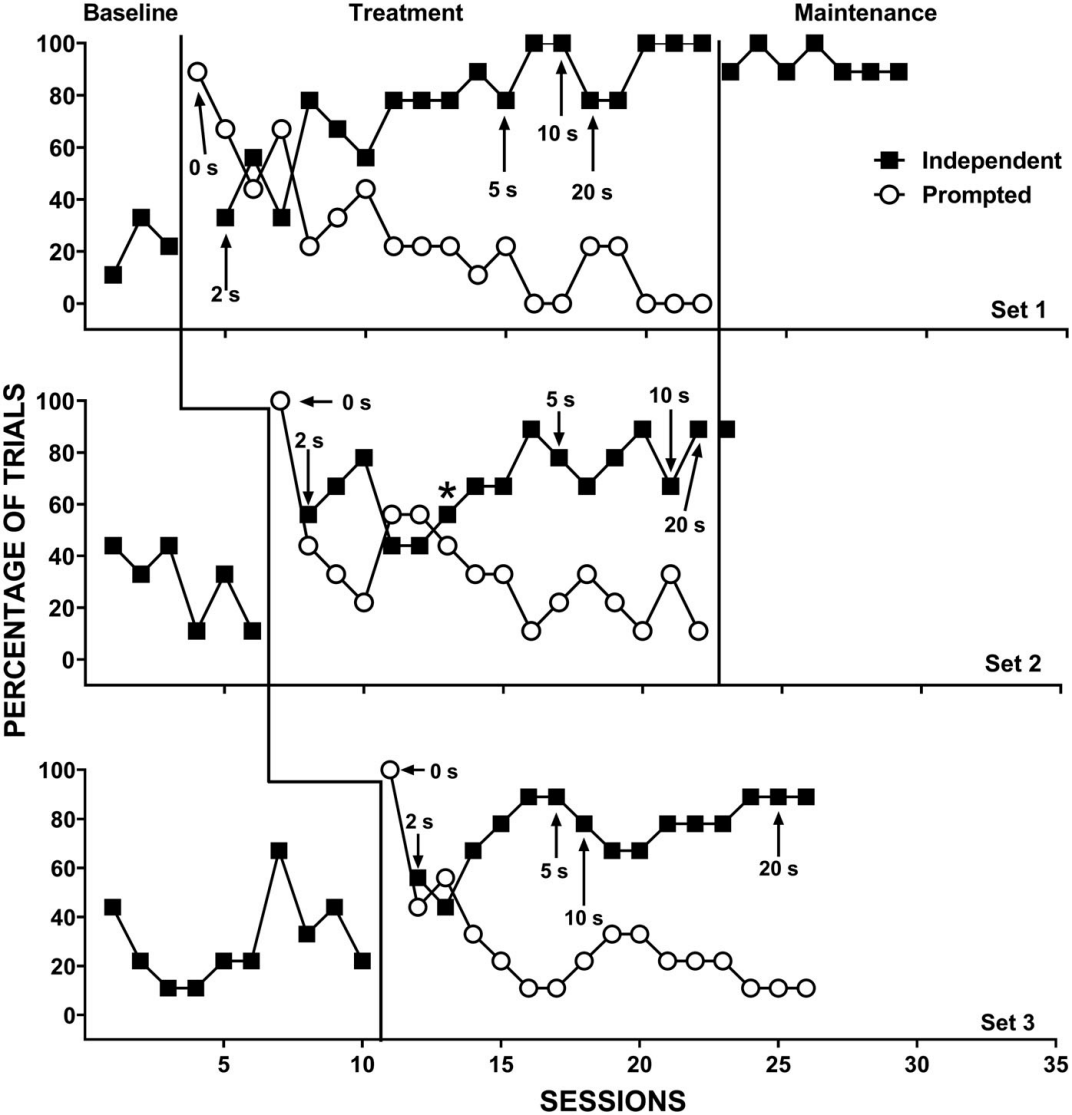
Independent correct responses (closed squares) and prompted correct responses (open circles) for Maria with Set 1 (top panel), Set 2 (middle panel), and Set 3 (bottom panel). The asterisk denotes the point at which we emphasized the difference between the verbal S^D^s “neck” and “desk”.

**Figure 2:**
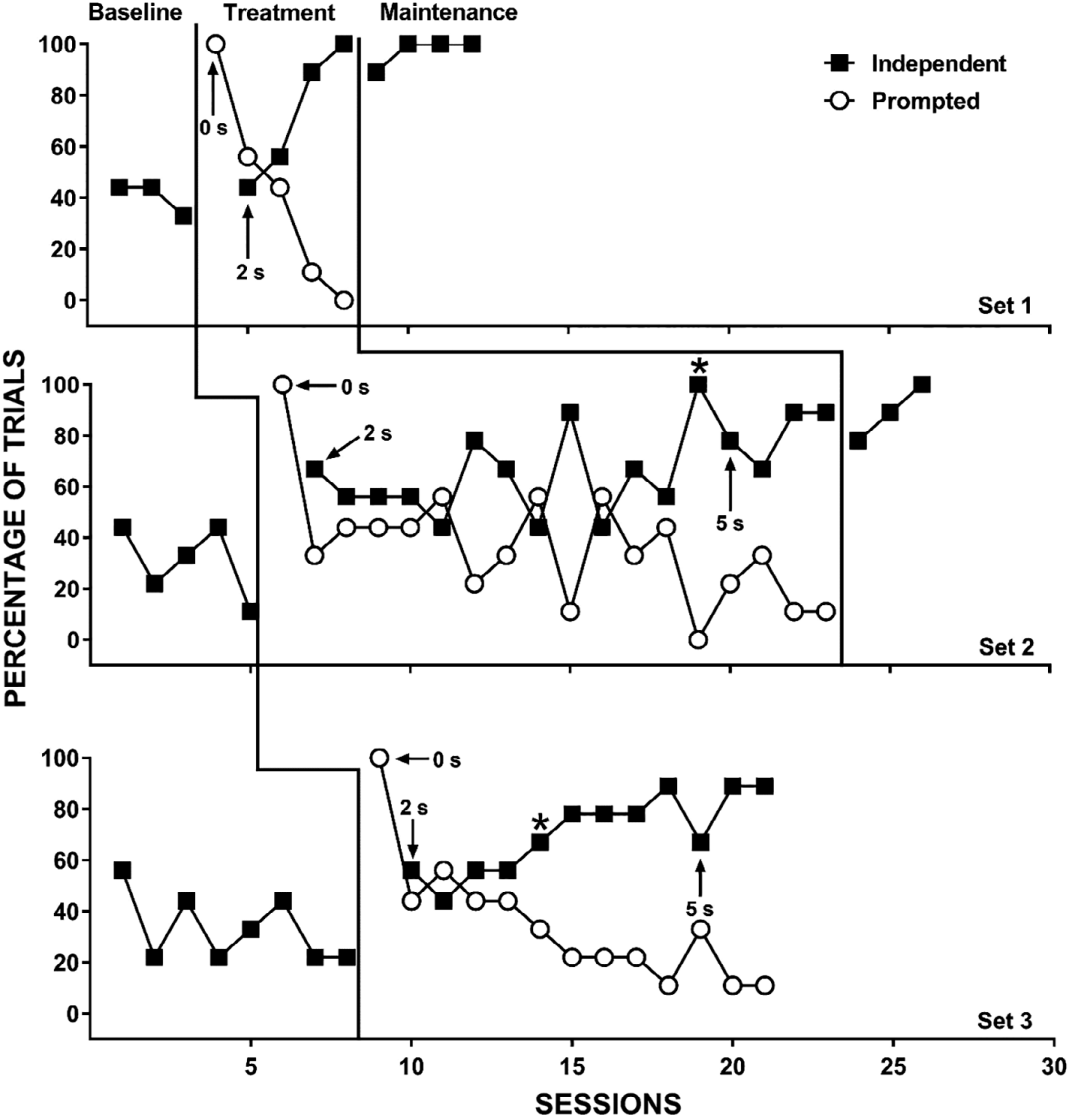
Independent correct responses (closed squares) and prompted correct responses (open circles) for Pierre with Set 1 (top panel), Set 2 (middle panel), and Set 3 (bottom panel). The asterisks denote the points at which we began requiring independent scanning.

**Figure 3:**
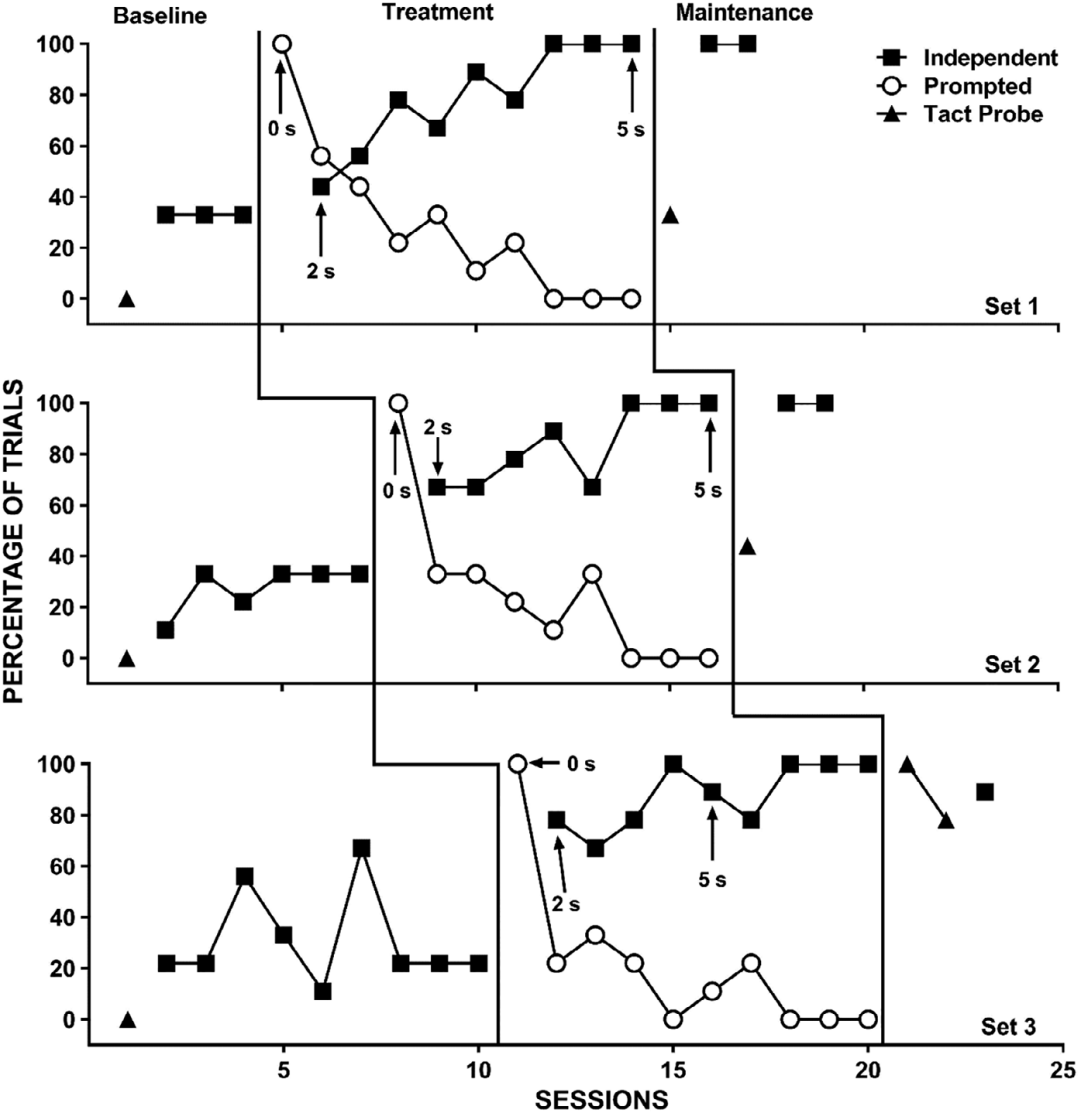
Independent correct responses (closed squares), prompted correct responses (open circles), and correct responses during tact probes (closed triangles) for Marco with Set 1 (top panel), Set 2 (middle panel), and Set 3 (bottom panel).

**Figure 4:**
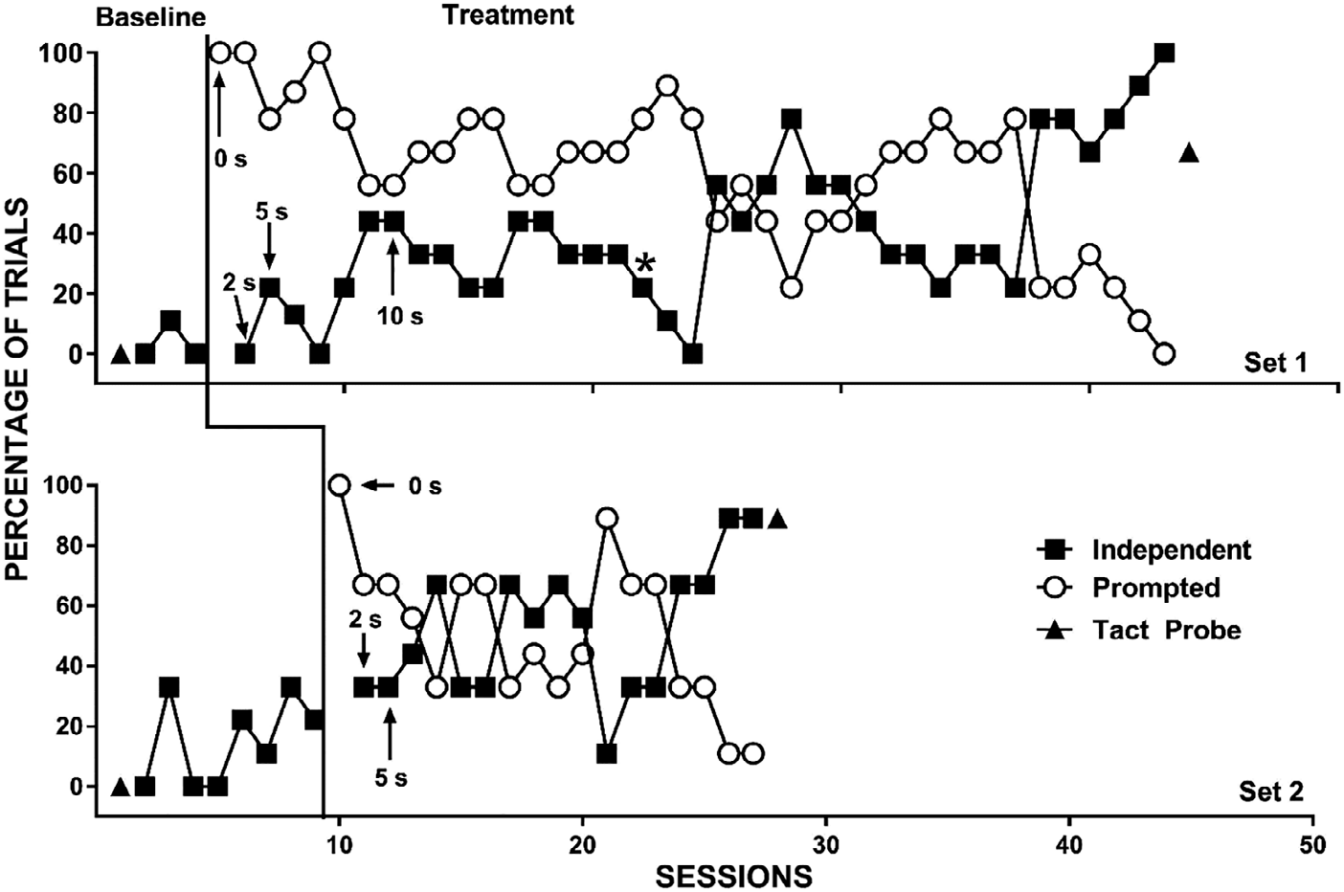
Independent correct responses (closed squares), prompted correct responses (open circles), and correct responses during tact probes (closed triangles) for Colton with Set 1 (top panel) and Set 2 (bottom panel). The asterisk denotes the point at which we addressed Colton's middle‐location bias.

The therapist provided reinforcement for correct prompted responses until the participant independently engaged in the correct response for at least four trials (about 44%) in one session. After this, the therapist only provided reinforcement following independent correct responses. If the participant did not engage in a prompted correct response within 5 s of the identity‐match prompt, the therapist physically guided the correct response (which occurred rarely during the study). The therapist then removed the comparison stimuli and re‐presented them with the same vocal S^D^ (i.e., the same trial) until the participant engaged in an independent correct response or the therapist re‐presented a given trial five times, whichever came first. During error‐correction trials, the therapist used the same prompting procedures as the initial trial for the echoic DOR and initially provided reinforcement for both prompted correct and independent correct responses (i.e., initially, more than one reinforcement interval occurred on trials without an initial independent correct response). Once the therapist removed reinforcement for prompted correct responses during the initial trial, the therapist also discontinued reinforcement for prompted responses during error‐correction trials. Only the first presentation (i.e., prior to any error‐correction procedures) during a given trial counted for the data presented in Figures 1, 2, 3, 4. That is, observers scored the trial as independent, prompted, or incorrect based on the first presentation of each of the nine trials in a session. However, we collected data on the number of re‐presentation trials required during each of the nine trials.

We implemented a progressive‐prompt delay to transfer stimulus control from the identity‐match prompt to the vocal S^D^ using the following progression: 0 s, 2 s, 5 s, 10 s, and 20 s. All participants received one session with a 0‐s prompt delay prior to moving to a 2‐s prompt delay and progressively increasing delays following each session in which the participant either selected the correct comparison stimulus or waited for the identity‐match prompt on at least eight of nine (89%) trials. The treatment phase ended when the participant met the mastery criterion which was at least two consecutive sessions with the percentage of independent correct responses at or above eight out of nine correct (i.e., 89%). In some cases, we extended the teaching phase until three consecutive sessions at or above 89% were observed, and for Set 2 with Maria the treatment phase was ended following only one session at 89% (due to therapist error).

#### *Maintenance*

After a participant met the mastery criterion in a set, we conducted maintenance sessions approximately once a week (except that we did not conduct maintenance sessions with Set 3 for Maria and Pierre, and we conducted no maintenance sessions with Colton due to his discharge from the clinic). We conducted maintenance sessions in a manner identical to baseline.

#### *Supplemental procedures for Pierre*

Pierre did not consistently and independently attend to each of the comparison stimuli in the array for Set 2 and Set 3 (i.e., scan the array). On trials in which Pierre did not scan the array prior to the vocal S^D^, he was less likely to respond correctly than when he did scan. Additionally, following the identity‐match prompt, Pierre almost always independently scanned the array and engaged in a prompted corrected response. Therefore, starting at Session 19 for Set 2 and Session 14 for Set 3, the therapist required independent scanning from Pierre before proceeding with each trial. If Pierre did not independently scan the array of comparison stimuli within 5 s, the therapist removed the comparison stimuli and turned away from Pierre for 5 s. After 5 s, the therapist re‐presented the comparison stimuli. The therapist continued this procedure until Pierre independently scanned the array, at which time the therapist presented the vocal S^D^. Pierre was the only participant for whom we did not observe consistent independent scanning of the array prior to the presentation of the vocal S^D^.

#### *Supplemental procedures for Colton*

Colton consistently selected the stimulus located in the middle position in the array, referred to as a positional bias (Grow & LeBlanc, 2013; Kangas & Branch, 2008). Therefore, beginning at Session 22 for Set 1, the therapist systematically manipulated the location of the target stimulus. Specifically, if Colton engaged in three consecutive incorrect responses to a specific target location (e.g., selected the middle stimulus, even though that stimulus was incorrect, three times in a row), the therapist would no longer place the correct comparison stimulus in that location until reinforcement had been provided for three correct independent responses. The therapist implemented these procedures not only for the initial trial but also for all error‐correction trials. The therapist returned to the original teaching procedures for Set 2.

### *Procedures Specific to Experiment 2*

For Marco and Colton, we conducted tact probes prior to initiating baseline and following treatment. During each tact probe, the therapist held up the target picture and said, “What is it?” The therapist presented each picture three times in each tact probe in a quasirandom and counterbalanced fashion. The therapist delivered no programmed consequences following correct or incorrect responses. However, the therapist provided reinforcement on a VR 2 schedule for other appropriate behaviors to maintain participant motivation.

### *Experiment 1 Results and Discussion*

Figure 1 displays Maria's percentage of trials with independent and prompted correct responding for Set 1 (top panel), Set 2 (middle panel), and Set 3 (bottom panel). During baseline, Maria showed low and variable levels of independent correct responses for all sets. Following the introduction of the treatment package, Maria's independent correct responding increased steadily across sessions for Sets 1 and 3. For Set 2, independent correct responding began to decrease at Session 11. After analyzing session data, we determined that Maria consistently made errors with the targets *neck* and *desk*. We noted that her echoic responses for these two targets sounded nearly identical, and we therefore hypothesized that she had difficulty differentiating between the two vocal S^D^s. Beginning with Session 13 (denoted by an asterisk in Figure 1), the therapist emphasized the difference between the targets when presenting the vocal S^D^. Specifically, the therapist elongated the word “desk” to make the “sk” sound more salient (i.e., dɛssssssk) and shortened the word “neck” to make the “ck” sound crisp and succinct (i.e., nɛk). We made these adjustments to increase the discriminability between these two sample stimuli and noted that Maria's echoic responses matched the form of the S^D^ presented by the therapist (i.e., once we elongated desk and shortened neck, Maria did as well). This change remained in place for the remainder of treatment and maintenance and Maria's percentage of independent correct responding increased and maintained at follow up.

Figure 2 displays Pierre's percentage of trials with independent and prompted correct responding for Set 1 (top panel), Set 2 (middle panel), and Set 3 (bottom panel). During baseline, Pierre displayed low and decreasing levels of independent correct responding across sets. Following the introduction of the treatment package, Pierre's independent responding increased to mastery for Set 1, but increased only slightly for Sets 2 and 3. Careful observation of Pierre during sessions led to procedural adjustments, as described in the previous section (denoted by the asterisks in Figure 2), which resulted in rapid acquisition of the Sets 2 and 3. His independent correct responding remained high during maintenance.

Figure 3 displays Marco's percentage of trials with independent and prompted correct responding for Set 1 (top panel), Set 2 (middle panel), and Set 3 (bottom panel). During baseline, Marco displayed low and stable levels of independent correct responding across sets except for Sessions 4 and 7 in Set 3, during which Marco selected the correct comparison stimulus on a moderate percentage of trials. Following the introduction of the treatment package, independent correct responding rapidly increased to high levels for all targets. His independent correct responding remained high during maintenance.

Figure 4 displays Colton's percentage of trials with independent and prompted correct responding for Set 1 (top panel) and Set 2 (bottom panel). During baseline, Colton engaged in low and stable levels of correct independent responding. When we introduced treatment in Set 1, we observed a slight increase in independent correct responding; however, independent correct responding never exceeded 44%. The trial‐by‐trial data (not shown) suggested that Colton had a middle position bias (i.e., he almost always selected the comparison stimulus located in the middle position regardless of the sample stimulus). Therefore, we introduced the position modification described above at Session 22 (denoted with an asterisk in Figure 4). Thereafter, the percentage of trials with correct independent responding decreased, then gradually increased, then decreased again, then finally increased to mastery level. When we introduced treatment in Set 2, we observed a more rapid increase in correct independent responding compared to Set 1.

### *Experiment 2 Results and Discussion*

Figures 3 and 4 also show the levels of correct tacts during the tact probes during baseline and after treatment with Marco and Colton. During baseline, neither Marco nor Colton displayed correct tacts for any of the stimuli in any of the sets. Following auditory‐visual conditional discrimination training, Marco's correct tacts increased progressively with each training set: 33% for Set 1, 44% for Set 2, and 89% for Set 3. He showed mastery‐level performance (correct tacts during three of three trials during the initial probe session following treatment) for five of the nine targets.

We observed a similar pattern with Colton; however, we were only able to complete two sets prior to his discharge from clinic. He displayed 67% correct tacts for Set 1 and 89% correct tacts for Set 2. Colton showed mastery performance for four of the six targets and correctly tacted a fifth target on two of three (67%) trials.

### *Post Hoc Results and Discussion*

We conducted several post hoc analyses following the completion of Studies 1 and 2. First, we compared the mean number of trials (including repetitions) required to master a set of targets in the current study to the mean number reported in other similar studies in the literature. Across the four participants and 11 sets of mastered stimuli, the current treatment package, with modifications for three participants, produced mastery performance across all 11 target sets (42 individual targets mastered in total; 33 listener targets and 9 tact targets). We only considered a tact target mastered if the participant correctly tacted the stimulus on every trial of the initial posttreatment probe. These outcomes appear to be more consistent and positive than the results of auditory‐visual conditional‐discrimination training in the published literature for individuals classified as novice learners with ASD (Carp et al., 2012; Kodak et al., 2011). For our participants, the mean number of trials to mastery, including the original and all repeated trials, equaled 218.4. This rate of acquisition is similar to results obtained with children with ASD who did not meet our criteria for novice learners. For example, Carroll et al. (2015) compared efficiency across a number of procedural variations for teaching auditory‐visual conditional discriminations (*M* = 204.6 trials to mastery for their most efficient procedure). These data suggest that the treatment package we developed and evaluated in this investigation, with learner‐specific modifications for three participants, appears to be a fairly effective and efficient approach to teaching initial auditory‐visual conditional discriminations to novice learners with ASD.

Next, we also examined within‐trial data to provide preliminary results regarding which of the components of the treatment package may have contributed to the observed effects. To do so, we examined how often the therapist implemented each of the components of the treatment package (except for the trial format, which did not vary across trials). Marco emitted echoics on 100% of trials for all three training sets; Maria emitted echoics for 99% of trials for Set 1 and 100% for Sets 2 and 3; Colton emitted echoics for 96% of trials for Set 1 and 100% of trials for Set 2. By contrast, Pierre emitted echoics on just 18% of trials for Set 1, 15% for Set 2, and 21% for Set 3. Pierre's data suggest that having him echo each sample stimulus may not have been a necessary component of the treatment package, because he mastered all three target sets despite infrequently overtly echoing the sample stimulus.

All four participants uniformly responded with prompted correct responses in the presence of the identity‐match prompt (*M* = 100%, 100%, 99.9%, and 99.5% for Pierre, Marco, Maria, and Colton respectively), indicating that it was an effective controlling prompt. More importantly, we also calculated the percentage of error‐correction trials in which the participant emitted an independent correct response on the first repetition of the trial. If an individual frequently displayed an independent correct response on this first re‐presentation, it would suggest that the identity‐match prompt increased the probability of a subsequent independent correct response. In fact, three of the four participants (Maria, Pierre, and Marco) emitted an independent correct response on the first repetition of a trial for 84.9% of error‐correction trials (range 61.7% to 100% across training sets). These results suggest that, for these participants, the identity‐match prompt produced an immediate increase in independent correct responses and probably helped to facilitate acquisition of the auditory‐visual conditional discriminations. Colton only emitted an independent correct response on the first repetition of a trial for 21% of the error‐correction trials during Set 1, although this percentage increased to 66% during Set 2. It may be that with additional exposure, the procedure became more efficacious; however, it may also be that the modification introduced during Set 1 to reduce biased responding may have altered the efficacy of this prompt.

## GENERAL DISCUSSION

Our primary goal for this investigation was to develop and evaluate a treatment package for establishing initial auditory‐visual conditional discrimination in novice learners with ASD, defined as individuals who had not previously mastered such discriminations and who received a standard score of 20 on the PPVT‐4. In addition, all participants scored at chance levels on Level 1 of the PPVT‐4. The treatment package included: (a) presenting learning trials in the conditional‐only trial format (Grow et al., 2011, 2014); (b) prompting the participant to echo the dictated sample stimulus as a DOR before allowing a selection response (e.g., Dube & McIlvane, 1999); (c) using a progressively delayed prompting procedure to transfer stimulus control from the controlling prompt to each sample stimulus (e.g., Grow & LeBlanc, 2013); (d) using an identity‐match prompt as both the controlling prompt for the selection response and to ensure that the individual discriminated the correct comparison stimulus from the others (e.g., Fisher et al., 2007); and (e) repeating trials contingent on errors until the participant emitted an independent correct response (e.g., Cariveau, La Cruz Montilla, Gonzalez, & Ball, 2018; Schumaker & Sherman, 1970). Four novice learners with ASD acquired initial auditory‐visual conditional discriminations using this training package, although three of the participants required learner‐specific modifications to the treatment.

It is important to note that although our participants had not previously mastered auditory‐visual conditional discriminations (i.e., were novice learners in this area), each participant could engage in echoic behavior and had mastered visual identity matching. These skills were necessary for the participant to engage in the DOR and respond to the identity‐match prompt. Therefore, it is unlikely that our treatment package, with or without modifications, would be effective for learners without these skills. Other research has suggested that individuals who do not master echoic programs may be less likely to acquire auditory‐visual conditional discriminations (Kodak et al., 2011, 2015); therefore, we recommend clinicians teach echoics and visual identity matching prior to initiating auditory‐visual conditional discrimination training. For example, Colton required teaching of both prerequisites prior to his participation in the project.

Although the treatment package proved sufficient for one participant, Pierre, Maria, and Colton required procedural modifications to the treatment package. Pierre required the addition of a procedure to promote independent scanning for Sets 2 and 3; Maria required slight modifications of the pronunciation of the vocal sample stimulus to meet mastery with Set 2; and Colton required an adjustment to the location of the correct comparison stimulus to address a middle‐location bias during Set 1 (e.g., Grow & LeBlanc, 2013). These adjustments suggest that some novice learners may require additional supports to master initial auditory‐visual conditional discriminations with our treatment package.

It is worth noting, however, that the initial DOR and identity‐prompt components of the treatment package facilitated our identification of the specific procedural adjustments required for Pierre and Maria. Requiring an echoic DOR for the sample stimulus facilitated our identification of the adjustment for Set 2 with Maria. With Pierre, we observed that he readily scanned the comparison stimuli following the presentation of the identity‐match prompt (and thereafter emitted a correct prompted response on 100% of trials), but did not do so prior to the presentation of the vocal sample stimulus. Thus, including a controlling prompt that ensured that participants attended to and discriminated the comparison stimuli facilitated our identification of the adjustment we made for Sets 2 and 3 with Pierre. Namely, we required that Pierre scan the comparison stimuli prior to the vocal S^D^ just as he did after the presentation of the identity‐match prompt. Another potential solution to Pierre's poor scanning may have been to use a sample‐first procedure. Petursdottir and Aguilar (2016) found faster acquisition when they presented the sample stimulus prior to the comparison stimuli with learners who showed typical development. Other research has found the sample‐first procedure to be more effective for the majority of participants (Cubicciotti, Vladescu, Reeve, Carroll, & Schnell, 2018; Schneider, Devine, Aguilar, & Pettursdottir, 2018); however, the participants in each of these studies did not meet our criteria for novice learners relative to acquisition of auditory‐visual conditional discriminations. Future research should compare a sample‐first procedure with the comparison‐first procedure with novice learners while holding other aspects of the treatment package constant.

Our secondary goal for this investigation was to provide a preliminary evaluation as to whether our treatment package could promote the emergence of tacts corresponding to the targets established as listener skills (e.g., tact “apple” when presented a picture of an apple after learning to select an apple in response to the spoken word “apple”; Miguel & Petursdottir, 2009; Petursdottir & Carr, 2011). Therefore, in Experiment 2, we assessed for the emergence of tacts during probes conducted in baseline and after treatment with Marco and Colton. Both Marco and Colton correctly tacted at least some stimuli following auditory‐visual conditional discrimination training, and the number of correctly tacted stimuli increased with each training set.

It is important to note that neither Marco nor Colton displayed a tact repertoire prior to this study, and both participants exhibited their first recognizable tacts as emergent responses following listener training. This finding appears to be unique in the behavior‐analytic literature, as a diligent search of the literature failed to find any cases in which the initial tacts of a child with ASD (or other developmental disability) emerged to this degree following listener training. In fact, although a full review of this literature is beyond the scope of this discussion, even among children with ASD with preexisting listener and tact repertoires, researchers have generally found inconsistent transfer from listener to speaker responding (Bao, Sweatt, Lechago, & Antal, 2017; Delfs et al., 2014; Ingvarsson, Cammilleri, & Macias, 2012; Lechago, Carr, Kisamore, & Grow, 2015; Petursdottir & Carr, 2011; Sprinkle & Miguel, 2012; but see DeSouza, Fisher, & Rodriguez, 2019, and Kobari‐Wright & Miguel, 2014, for notable exceptions). Future research should attempt to replicate the procedures used with Marco and Colton to determine the generality of our preliminary findings.

We speculate that, consistent with the naming hypothesis (Horne & Lowe, 1996), our treatment package promoted the emergence of initial tacts following listener training for Marco and Colton because we required them to echo the name of the sample stimulus and then attend to the visual comparison stimuli via the identity‐match prompt followed by reinforcement. Requiring the child to listen to and then echo the name of a stimulus while attending to a picture of that stimulus increases the likelihood that the child will simultaneously emit interrelated speaker and listener behavior followed by reinforcement (Contreras, Cooper, & Kahng, 2019; Miguel, 2018). According to Horne and Lowe (1996), bidirectional emergent relations emanate from the child simultaneously behaving as a speaker and a listener; for example saying, “car,” and hearing oneself say, “car,” while looking at a car facilitates the emergence of novel responding, such as the emergence of tacts following listener training.

There are procedural modifications and limitations worth noting in our study. First, we did not apply a consistent mastery criterion across participants. Future researchers should standardize the mastery criterion to ensure a standard metric across participants. Second, we provided reinforcement for correct prompted responses, during both the initial trial and error‐correction trials, until the participant engaged in correct independent responses on at least 44% of trials for one session. We provided reinforcement for prompted responses initially to avoid the participants experiencing lengthy periods without reinforcement, which might have evoked untoward side effects. Although it did not appear to be the case with any of our participants, reinforcing prompted correct responses could result in the participant learning to make errors in order to increase the frequency of reinforcement during a session. Future research should examine differences in acquisition when experimenters initially reinforce both prompted and independent correct responses compared to when they provide reinforcement only for independent correct responses.

Finally, we extended the time delay between the initial presentation of the sample stimulus and the identity‐match prompt to 20 s for Maria. Most researchers using progressive prompting procedures have placed a lower ceiling on the duration of the delay (e.g., 10 s; Walker, 2008). However, the original article that formed the basis for most time‐delay procedures used a 15‐s delay to good success (Halle, Marshall, & Spradlin, 1979). In addition, there is not a clear reason for setting a ceiling on the delay, because a main purpose of the delay is to increase the establishing operation for the target response through the passage of time. Thus, for someone who is not making errors of commission and who consistently waits for the controlling prompt, extending the delay to the prompt may be reasonable.
